# Ultramicronized Palmitoylethanolamide in the Management of Neuropathic Pain Related to Chronic Inflammatory Demyelinating Polyneuropathy: A Proof-of-Concept Study

**DOI:** 10.3390/jcm13102787

**Published:** 2024-05-09

**Authors:** Dario Cocito, Erdita Peci, Maria Claudia Torrieri, Marinella Clerico

**Affiliations:** 1 Clinical and Biological Sciences Department, University of Turin, 10043 Orbassano, Italy; 2 Academic Neurology Unit, San Luigi Gonzaga University Hospital, 10043 Orbassano, Italy; 3 Academic Neurology Unit, San Luigi Gonzaga University Hospital, Clinical and Biological Sciences Department, University of Turin, 10043 Orbassano, Italy

**Keywords:** ultramicronized palmitoylethanolamide, neuropathic pain, immunoglobulins, chronic inflammatory demyelinating polyneuropathy, autoimmune disease

## Abstract

**Background/Objectives**: Chronic inflammatory demyelinating polyneuropathy (CIDP) is a rare autoimmune disease. Neuropathic pain (NP), related to peripheral inflammation, is among its earliest manifestations. This preliminary open-label investigation aimed to evaluate the efficacy of ultramicronized Palmitoylethanolamide (umPEA) in the management of NP. **Methods**: A total of 14 patients with CIDP, already undergoing immunoglobulin (Ig) therapy, were divided into two groups: Group A received umPEA 600 mg twice daily in addition to Ig for 60 days, followed by Ig alone until the end of the observation (180 days); Group B received Ig alone for 120 days and subsequently umPEA + Ig in the last 60 days of the study. Painful symptom intensity and quality of life were assessed by the Numeric Rating Scale, Neuropathic Pain Symptoms Inventory, and Five Dimensions Health Questionnaire. The safety umPEA profile was evaluated. **Results**: UmPEA in addition to immunoglobulins allowed for a significant improvement over time in all NP symptoms intensity (*p* = 0.0007) and in patients’ quality of life (*p* = 0.0036). **Conclusions**: This study suggests umPEA as a safe and effective treatment in addition to immunoglobulins to improve NP, ameliorating the patient’s health status. These results highlight the importance of neuroinflammation modulation in the management of CIDP’s painful symptoms, drawing attention to umPEA’s potential use also in neuropathies of different etiologies.

## 1. Introduction

Chronic inflammatory demyelinating polyneuropathy (CIDP) is defined as a rare relapsing/remitting or progressive autoimmune neuropathy, characterized by a multifaceted presentation, still unknown etiology, and partially understood pathophysiology [1], which includes several humoral and cell-mediated mechanisms [2,3]. Despite macrophage-induced demyelination being crucial for the pathogenesis of CIDP, recent evidence has also indicated the presence of distinctive mechanisms initiated by autoantibodies against para-nodal junction proteins [4,5]. The peripheral nerve demyelination, caused by the aberrant immune response, manifests as progressive weakness, numbness, paresthesia, sensory ataxia, fatigue, imbalance, neuropathic pain (NP), and impaired ambulation, leading to substantial disability. As the disease progresses, axonal damage of peripheral nerves occurs secondary to demyelination, which is associated with further disease progression and the subsequent worsening of symptoms [2,6,7].

The care for patients affected by CIDP is complex and requires individualized strategies. The first-line therapies recommended for patients with moderate to severe disability include corticosteroids and intravenous immunoglobulins (IVIg), while plasma exchange and a combination of either immunosuppressants or immunomodulators should be considered when IVIg and corticosteroids are ineffective or in case the response to the individual treatments are inadequate or the drug maintenance dose is high [8,9,10]. Such therapies, while essential, are associated with adverse events or reduced patient independence: corticosteroids are correlated with long-term tolerability, and IVIg should be regularly administered at home under nurse supervision or in a clinical setting [7]. Although immunoglobulin therapy is commonly administered intravenously, recent evidence has shown that the subcutaneous administration offers comparable efficacy and various advantages, such as optimal tolerability due to the mild-to-moderate transient local side reactions, over the IVIg systemic adverse events and an easier route of administration, thus providing benefits and improving patients’ quality of life [11,12].

Among the symptoms experienced by patients with CIDP, one is NP, clearly related to the inflammation of peripheral nerves and spinal roots [13]. In general, immune monotherapy treatment is sufficient to manage CIDP neuropathic pain, but specific adjuvant therapies are often necessary when pain persists [13,14]. The pharmacological treatment for NP considers drugs recommended by published guidelines (Good Practice Point) and includes tricyclic antidepressants, pregabalin, gabapentin, or serotonin-noradrenaline reuptake inhibitors such as duloxetine or venlafaxine. These drugs, in addition to being obviously related to different adverse effects, are generally used for the maintenance treatment of CIDP [10].

It is recognized that the chronic inflammatory processes sustaining NP are countered by a resolution program that involves the production of lipid mediators able to turn off inflammation. N-acylethanolamines, a class of naturally anti-inflammatory and pro-resolving mediators, may thus represent new therapeutic opportunities for chronic pain management. Palmitoylethanolamide (PEA), in particular, is widely distributed in different tissues, especially nervous ones [15], and is synthesized ‘on demand’ during inflammatory and neurodegenerative conditions to oppose inflammation, pain, and neuronal damage [16]. Endogenous PEA, whose levels are altered as a result of stress or injury and/or pain, accumulates in tissues and acts via the ALIA (Autacoid, Local, Injury Antagonism) mechanism, down-modulating mast cells and microglia hyperactivity, protecting neurons against excitotoxicity, reducing tissue inflammation, and exerting neuroprotective functions [17,18]. It is known that mast cells and microglia have a crucial role in the development and maintenance of neuropathic pain: when activated, mast cells release histamine, tumor necrosis factor alpha (TNFα), and nerve growth factor (NGF), which can sensitize nociceptors, while microglia releases cytokines, prostaglandins, and amino acids, contributing to neuronal excitability and pain onset [19]. Besides these mechanisms, PEA also elicits its anti-inflammatory and pain-relieving effects by the action of different receptors located on the nociceptive pathway [20]. PEA, in fact, activates the nuclear peroxisome proliferator-activated receptor alpha (PPAR-α) and the orphan receptor G-protein coupling (GPR55), modulating both the perception and transmission of peripheral pain signaling. Moreover, PEA acts via the so-called ‘entourage effects’ by indirectly activating cannabinoid (CB2 and CB1) receptors or the transient receptor potential vanilloid receptor type-1 channels (TRPV1) [16,21,22,23,24,25,26].

The pain-relieving effect of PEA in its micronized (mPEA) and ultramicronized (umPEA) forms (which increase its bioavailability and biological efficacy) [27,28] was demonstrated in patients suffering from chronic pain of different origins, such as radiculopathy, osteoarthritis, postherpetic neuralgia, diabetic neuropathy, and pain associated with oncologic disease, suggesting an independence between umPEA’s effects and pain pathogenesis [29,30,31,32,33,34]. Importantly, it was also reported that m/umPEA, in addition to the lack of both acute and chronic toxicity, is well tolerated and has no interactions with other concomitant pharmacological therapies [16,35].

Based on the view that umPEA modulates mechanisms underlying different conditions associated with chronic neuropathic pain, we aimed to assess whether umPEA in addition to immunoglobulin (Ig) therapy could be an innovative approach for the treatment of the painful symptoms associated with CIDP, thus improving the health status of patients suffering from this disease.

## 2. Materials and Methods

### 2.1. Study Participants

This proof-of-concept investigation was carried out at the Clinical and Biological Sciences Department, AOU San Luigi Gonzaga in Orbassano (Italy), in 14 outpatients suffering from CIDP and undergoing intravenous (IV) or subcutaneous (SC) immunoglobulin therapy. Patients, afferent to the Department from 30 January 2023 to 30 January 2024 for the planned immunoglobulin therapy, were consecutively considered. 

Inclusion criteria were as follows: (a) both sexes; (b) age ≥ 18 years; (c) CIPD diagnosis according to the European Federation of Neurological Societies and the Peripheral Nerve Society (EFNS/PNS) criteria; (d) treatments with immunoglobulins (≥4 doses) upon the 9 months preceding the enrolment visit; (e) treatment with IV or SC immunoglobulins at least 8 weeks before the eligibility screen; (f) score ≥ 4 on Douleur Neuropathique 4 questionnaire (DN4) indicative of neuropathic pain; (g) pain intensity score ≥ 5 on the Numeric Rating Scale (NRS). 

Patients with polyneuropathy due to other causes or with concomitant disease, which could interfere with the evaluations, were excluded from the study. 

Written informed consent was obtained from all patients considered for our investigation. The study was conducted in accordance with the Declaration of Helsinki with regard to ethical principles for medical research involving human subjects and according to the Good Clinical Practice (GCP). The publication of the observed data was authorized by the Clinical and Biological Sciences Department Scientific Board of San Luigi Gonzaga Hospital in Orbassano.

### 2.2. Study Design

This preliminary open-label study tested the effect of umPEA (Normast 600^®^, Epitech Group SpA, Saccolongo, Padova, Italy, available on the market for neuropathic pain treatment) in addition to immunoglobulins, compared to immunoglobulin therapy alone without the addition of placebo. 

All patients were individually observed every month for 180 days, adopting the N-of-1 approach [36,37,38] to evaluate the individual response to treatment.

UmPEA supplementation consisted of umPEA 600 mg twice daily for 60 days, according to the available literature data [29,32,39]. The existing therapy with IV or SC immunoglobulins was continued throughout the observation period, without any suspension or variation, in order to minimize bias.

**Figure 1:**
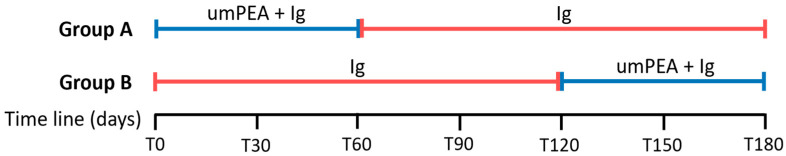
Treatment scheme and evaluation time points.

Eligible patients were randomized into two groups (A and B), regardless of the type of immunomodulatory therapy (IVig or SCIg), using a computer-generated randomization list. Group A was composed of patients who began umPEA in addition to Ig at the time of enrollment, taking it for 60 days, and then continuing the treatment with only immunoglobulins for the subsequent 120 days; Group B included patients who performed an initial period with Ig alone and subsequently began the treatment with umPEA in addition to Ig therapy (Figure 1).

### 2.3. Outcomes

All patients were subjected to the following assessments at enrolment (T0), after 30 (T30), 60 (T60), 90 (T90), 120 (T120), 150 (T150), and 180 days (T180) to evaluate:

(i) The presence of NP, evaluated at T0 by the DN4 questionnaire consisting of 10 items grouped into four questions of which seven concern pain description (burning, painful cold, electric shocks) and its associated sensations (tingling, pins and needles, numbness, itching), and the other three relate to a neurological examination in the painful area (touch hypoesthesia or pinprick hypoesthesia and tactile dynamic allodynia, clinically examined using a needle, von Frey filaments, and a standardized brush, respectively). The score of each item is 1 if yes, 0 if no. The resulting final score ranges from 0 to 10, with a cut-off value for diagnosis of neuropathic pain ≥ 4 [40];
(ii) The pain intensity, assessed at T0, T30, T60, T90, T120, T150, and T180 by the 11-point NRS, scored from 0 to 10, where 0 indicates ‘no pain’ and 10 corresponds to ‘the worst pain ever possible’ [41];
(iii) The symptoms and intensity of NP, evaluated at each time point by the Neuropathic Pain Symptoms Inventory (NPSI), consisting of 12 items: ten descriptors of the different symptoms and two elements to evaluate the duration of spontaneous ongoing and paroxysmal pain. For each item, a score of 0 indicates the best situation, while a score of 10 corresponds to the worst condition. NPSI subscores were also evaluated separately to assess burning superficial spontaneous pain (BSSP), pressing deep spontaneous pain (PDSP), paroxysmal pain (PP), evoked pain (EP), and paresthesia/dysesthesia (PD), corresponding to the average scores of the items belonging to each of the five pain factors [42];
(iv) The quality of life, evaluated at each time point by the five-dimension health questionnaire for quality of life (EQ5D) consisting of: (a) five items assessing health aspects such as mobility, self-care, activities, pain/discomfort, and anxiety/depression, with a score of 1 (‘no problems’), 2 (‘some problems’), or 3 (‘extreme problems’), resulting in a health profile; (b) the visual analogue scale (VAS), which allows the patient to describe his general state of health with a score ranging from 0, indicating the ‘worst imaginable health state’, to 100, which represents ‘the best imaginable health state’ [43].

### 2.4. Safety Assessments

All patients underwent routine blood analyses (at T0 and T60 for patients of Group A, at T120 and T180 for patients of Group B) and were monitored for any possible occurrence of adverse events related to umPEA intake throughout the entire course of the study.

### 2.5. Statistical Analysis

Data analysis was conducted using the Generalized Linear Mixed Model (GLMM). The distribution of patients by gender was evaluated using the Fisher test; age, DN4, NRS, and NPSI scores at enrolment were compared using Student’s *t*-test. Variables such as gender and age were included in the model as covariates. Values are expressed as mean ± standard error (S.E.) or standard deviation (S.D.), as specified. A *p*-value of less than 0.05 was considered statistically significant. The statistical analysis was performed using SAS 9.4 software.

## 3. Results

### 3.1. Baseline Characteristics of Patients

Fourteen patients (7 females and 7 males), aged (±S.D.) 56.3 (±16.5) years old, were enrolled. All patients completed the study periods with 100% treatment adherence. 

At baseline, all the patients had a score indicative of neuropathic pain (≥4) on the DN4 questionnaire, resulting in an average DN4 score of 6.4 ± 1.22 (Table 1).

Group A and Group B were homogeneous in terms of the number of female/male participants, patients’ mean age, and average pain intensity and symptoms (Table 1).

### 3.2. Pain Intensity (NRS)

**Figure 2:**
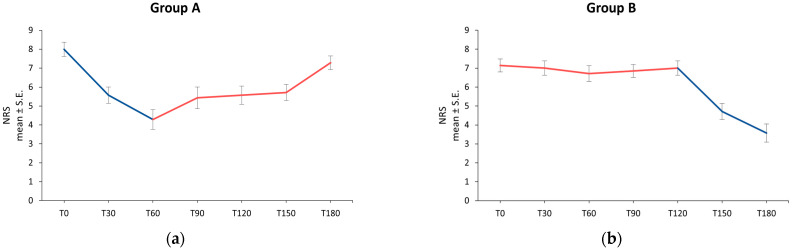
Pain intensity over time in Group A (**a**) and Group B (**b**); ── umPEA + Ig; ── Ig. NRS: Numeric Rating Scale; S.E.: Standard Error; T: Time point (days).

Among the Group A patients, in the 60 days of umPEA + Ig treatment, the severity of pain improved during the observation period: the average NRS score decreased from 8.0 ± 0.38 at T0 to 5.6 ± 0.43 at T30 and to 4.3 ± 0.52 at T60. During the subsequent period, when patients received only immunoglobulin therapy, a slight increase in the average NRS score was observed. The NRS mean value of 5.4 ± 0.57 at T90 increased to 5.6 ± 0.48 at T120, 5.7 ± 0.42 at T150, and to 7.3 ± 0.36 at T180 (Figure 2a). 

In patients of Group B, the NRS mean score remained stationary from T0 to T120 (7.1 ± 0.34 at baseline, 7.0 ± 0.38 at T30, 6.7 ± 0.42 at T60, 6.9 ± 0.34 at T90, 7.0 ± 0.38 at T120), and decreased from the 120th day of observation, corresponding to the beginning of umPEA supplementation, reaching an average NRS value of 4.7 ± 0.42 at T150 and 3.6 ± 0.48 at the end of the treatment (Figure 2b). 

The GLMM statistical analysis showed the umPEA efficacy over time: the pain intensity evaluated by NRS score showed a significant improvement over time, with a statistically significant difference between the therapy with immunoglobulins alone and the treatment with umPEA + Ig, in favor of umPEA supplementation (*p* = 0.0015).

### 3.3. Neuropathic Pain Symptoms and Intensity (NPSI)

**Figure 3:**
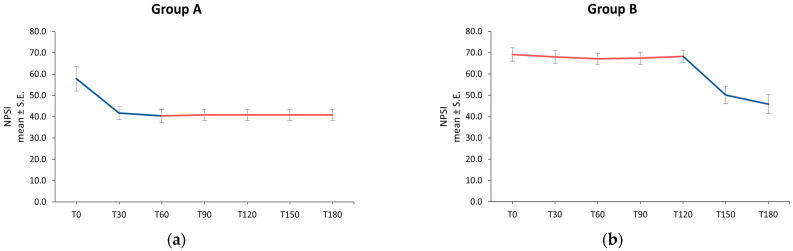
NPSI score over time in Group A (**a**) and Group B (**b**); ── umPEA + Ig; ── Ig. NPSI: Neuropathic Pain Symptoms Inventory; S.E.: Standard Error; T: Time point (days).

In Group A patients, the intensity of the neuropathic pain evaluated by NPSI showed an improvement from baseline (NPSI score 57.9 ± 5.80) to T30 (41.7 ± 2.96) and reached a mean value of 40.4 ± 3.15 at T60. During the following period, when patients were treated only with Ig, the NPSI score detected at T90 (40.9 ± 2.53) was maintained until the end of the study (Figure 3a).

In Group B patients, the NPSI mean score appeared practically unchanged from T0 to T120: the mean values were 69.1 ± 3.12 at T0, 68.0 ± 3.00 at T30, 67.1 ± 2.57 at T60, 67.4 ± 2.84 at T90, and 68.3 ± 2.81 at T120. After starting treatment with umPEA in addition to Ig, the score decreased to a mean value of 50.1 ± 4.07 at T150 and to 45.9 ± 4.43 at T180 (Figure 3b).

The NPSI score improved significantly over time with a statistically significant difference between the therapy with immunoglobulins alone and the umPEA supplementation in addition to Ig, in favor of the umPEA + Ig treatment (*p* = 0.0007).

The NPSI subscore analysis revealed that all the neuropathic pain expressions improved over time, with a statistically significant difference between the umPEA + Ig treatment and the immunoglobulin therapy alone, in favor of umPEA + Ig supplementation. In particular, the most evident improvement was achieved in BSSP (*p* = 0.0010), PP (*p* = 0.0011), and PD symptoms (*p* = 0.0008) (Table 2).

### 3.4. Quality of Life (EQ5D)

**Figure 4:**
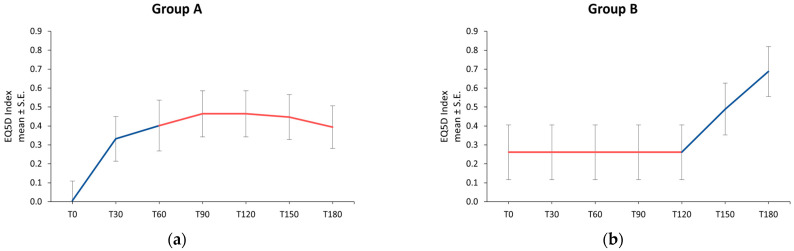
EQ5D Index over time in Group A (**a**) and Group B (**b**); ── umPEA + Ig; ── Ig. EQ5D: Five-dimension health questionnaire for quality of life; S.E.: Standard Error; T: Time point (days).

Quality of life evaluated by the EQ5D questionnaire showed an improvement over time in Group A patients: in the first 60-day treatment period with umPEA + Ig, the EQ5D Index score increased from 0.005 ± 0.104 at baseline to 0.332 ± 0.118 at the second time point and to 0.402 ± 0.134 at T60. At T90, the EQ5D score was 0.464 ± 0.121, which was maintained at T120 and slightly decreased to 0.447 ± 0.118 and to 0.394 ± 0.112 at T150 and T180, respectively (Figure 4a).

Patients in Group B reported an unchanged EQ5D score from T0 to T120 (0.262 ± 0.144), which then improved at T150 (0.489 ± 0.137) and improved even more at the end of the study period (0.687 ± 0.132) (Figure 4b).

Patients’ quality of life showed a statistically significant improvement over time during the umPEA + Ig-treatment period compared to the therapy with only immunoglobulins (*p* = 0.0036), demonstrating the efficacy of umPEA supplementation.

In both Group A and Group B patients, the performance of the EQ5D VAS score at the different evaluation time points was similar to that obtained in the EQ5D Index, as shown in Table 3.

### 3.5. Treatment Safety and Tolerability

No adverse events related to umPEA supplementation were observed over the course of the study. Routine blood analysis revealed no clinically relevant deviations from their normal values in relation to umPEA treatment.

### 3.6. Results Summary

These results, albeit within the limitations of the study, showed that umPEA, in association with the usual immunoglobulin therapy, improved the intensity of neuropathic pain symptoms and the quality of life of patients suffering from CIDP.

## 4. Discussion

Neuropathies are the most common type of peripheral nervous system disorder in adults, and approximately 50% of all neuropathies are associated with pain [44].

CIDP, in particular, presents as a progression of weakness over a period of at least 8 weeks, which in most cases evolves over the years, resulting in a severe neurological handicap, negatively impacting the health status of patients suffering from it [45]. Neuropathic pain, related to the inflammation of peripheral nerves and spinal roots and accompanied by other symptoms such as weakness and sensory loss, is among the first manifestations of the disease [13,46]. 

Only a few studies have described the specific management of pain in patients with CIDP and neuropathic pain. The NP treatment was described in a small number of patients and successfully treated with opioids, a combination of opioids, and amitriptyline or gabapentin [47,48]. From the perspective of finding new, effective, and safer approaches, this study investigated whether, in patients with CIDP, umPEA supplementation in addition to immunoglobulins can improve the intensity of NP symptoms more than immunoglobulin therapy alone, especially when painful symptoms continue to persist despite basic immunomodulatory therapy. During the 60 days of umPEA supplementation, patients of both Group A and Group B reported a significant improvement in pain intensity and in all NP symptoms. Notably, as shown by the NPSI subscores analysis, the umPEA treatment was particularly effective in the improvement of BSSP, PP, and paraesthesia/dysesthesia. 

Injuries or diseases of the somatosensory nervous system alter the function of primary sensory neurons and their central projection pathways, in addition to being associated with an immune response at every level of the somatosensory system. Initiating and persisting neuropathic pain involves communication between neurons and non-neuronal immunocompetent cells, such as mast cells and microglia, along with a cascade of pro- and anti-inflammatory mediators [27,49]. Microglia, which are normally in a ‘resting’ state in order to provide constant immune surveillance, following a trauma or a nerve injury, become activated. This circumstance is characterized by an increased expression of cell surface markers and receptors and by the release of algesic mediators [50,51]. Endogenous PEA exerts its pain-relieving effect, reducing the hyperalgesic component of neuropathic pain, precisely through the modulation of mast cells and microglia hyperactivity and by restoring the sensitization of peripheral nerves elicited by the release of inflammatory cytokines at the nerve injury site [52,53]. When stressful conditions, inflammatory stimuli, injury, or pain are protracted, endogenous PEA may be ‘depleted’. In such cases, the exogenous administration of this lipid mediator may become important to restore its protective, anti-inflammatory, and analgesic properties [54]. These effects were demonstrated both in chronic inflammation and neuropathic pain animal models, where PEA treatment resulted in pain perception control (by the modulation of mast cell activation), peripheral nerve morphology preservation, endoneural edema reduction, and a decrease in pro-inflammatory substance production at the injury site. Additionally, PEA prevented macrophage infiltration in the nerve and exerted anti-hyperalgesic and neuroprotective properties directly intervening in the nervous tissue alterations responsible for pain [19,55,56]. Furthermore, the exogenous supplementation of PEA has been shown to delay mast cell recruitment, protect mast cells against degranulation, and enhance the endogenous neuroprotective effect of PEA through the modulation of microglia activation [19].

CIDP, being a chronic disease, can lead to different functional complications of unpredictable duration and sequelae. In addition to physical disability, factors like pain, fatigue, anxiety, and depression may significantly affect the patient’s quality of life and also the course and outcome of the disease [57,58]. It is well known that neuropathic pain severity can impact patients’ quality of life more than functional disability, suggesting that its symptomatic management might be one of the most relevant targets in the treatment of peripheral neuropathies [59,60]. The results of this study suggest that the beneficial effect of umPEA as an add-on treatment in the control of neuropathic pain results also in an amelioration of the patient’s health-related quality of life. In addition, these results confirm the safety profile of umPEA, as no patients reported side effects. This represents an advantage over the currently available therapies and lays the foundation for considering this supplement as an appropriate alternative to manage the neuropathic pain associated with CIDP. 

This study, due to its preliminary nature, has some limitations, including the low number of patients, as well as the monocentric and open-label design. However, these promising data pave the way to better investigate the adjuvant treatment with umPEA as a new opportunity for patients suffering from CIDP who present not fully managed neuropathic pain.

For these reasons, further investigations with a double-blind, controlled, against-placebo design are needed to confirm the usefulness of umPEA in the management of CIDP neuropathic pain.

## 5. Conclusions

One of the most important problems of the therapy for chronic dysimmune neuropathy is the treatment of neuropathic pain, which is generally not controlled by the usual therapy to which patients suffering from CIDP are subjected. Such therapies, while improving the patient’s disability, do not improve their quality of life due to the persistent presence of pain. Our proof-of-concept study shows a possible way forward and an alternative to the current therapies that involve the use of opioids, antidepressants, or antiepileptics for the management of NP. The promising results of this preliminary study support, in fact, the over-time efficacy of umPEA, as an additional treatment to the standard Ig therapy, in the improvement of both the neuropathic pain and quality of life of patients with CIDP. These findings, highlighting the potential beneficial effects of umPEA that have also been demonstrated in other types of neuropathies, such as immune-mediated or diabetic neuropathies, further support its use to relieve neuropathic pain of various origins.

## Figures and Tables

**Table 1:** Patients baseline characteristics.

	Group A	Group B	All	*p*
Gender				1.0000
Female, n	3	4	7	
Male, n	4	3	7	
Age, mean years ± SD	58.0 ± 16.3	54.6 ± 17.8	56.3 ± 16.5	0.7140
DN4, mean score ± SD	6.1 ± 1.21	6.6 ± 1.27	6.4 ± 1.22	0.5314
NRS, mean score ± SD	8.0 ± 1.00	7.1 ± 0.90	7.6 ± 1.02	0.1176
NPSI, mean score ± SD	57.9 ± 15.35	69.1 ± 8.25	63.5 ± 13.21	0.1123

DN4: Douleur Neuropathique 4 questionnaire; NRS: Numeric Rating Scale; NPSI: Neuropathic Pain Symptoms Inventory; SD: Standard Deviation.

**Table 2:** The mean value of NPSI subscores at each time point.

NPSI Subscores	Group	T0	T30	T60	T90	T120	T150	T180	p umPEA + Ig vs. Ig
BSSP	Group A	7.3 ± 1.38	4.7 ± 1.08	4.4 ± 1.04	4.6 ± 1.04	4.6 ± 1.04	4.6± 1.04	4.6 ± 1.04	0.0010 *
	Group B	8.3 ± 0.42	8.0 ± 0.44	7.7 ± 0.29	7.9 ± 0.34	8.1 ± 0.26	5.4 ± 0.72	4.6 ± 0.78	
PDSP	Group A	4.4 ± 1.24	3.7 ± 1.07	3.6 ± 1.04	3.7 ± 1.04	3.7 ± 1.04	3.7 ± 1.04	3.7 ± 1.04	0.0138 *
	Group B	6.8 ± 0.82	6.6 ± 0.80	6.7 ± 0.82	6.7 ± 0.81	6.8 ± 0.84	6.1 ± 0.88	5.8 ± 0.87	
PP	Group A	6.6 ± 1.01	5.1 ± 0.90	4.9 ± 0.95	5.1 ± 0.94	5.1 ± 0.94	5.1 ± 0.94	5.1 ± 0.94	0.0011 *
	Group B	5.8 ± 0.86	5.6 ± 0.82	5.8 ± 0.86	5.7 ± 0.84	5.8 ± 0.86	4.2 ± 0.66	3.9 ± 0.67	
EP	Group A	5.1 ± 0.74	3.6 ± 0.50	3.5 ± 0.44	3.5 ± 0.47	3.5 ± 0.47	3.5 ± 0.47	3.5 ± 0.47	0.0045 *
	Group B	6.2 ± 0.53	6.1 ± 0.57	6.0 ± 0.45	6.2 ± 0.41	6.2 ± 0.50	4.3 ± 0.45	4.1 ± 0.49	
PD	Group A	6.6 ± 1.02	4.3 ± 0.87	4.2 ± 0.84	4.1 ± 0.78	4.1 ± 0.78	4.1 ± 0.78	4.1 ± 0.78	0.0008 *
	Group B	8.5 ± 0.29	8.5 ± 0.29	8.1± 0.21	8.0 ± 0.24	8.2 ± 0.18	5.6 ± 0.54	4.8 ± 0.72	

BSSP: Burning Superficial Spontaneous Pain; PDSP: Pressing Deep Spontaneous Pain; PP: Paroxysmal Pain; EP: Evoked Pain; PD: Paresthesia/Dysesthesia. * Significant change over time; *p* < 0.05.

**Table 3:** EQ5D VAS score at each time point.

	T0	T30	T60	T90	T120	T150	T180
Group A	56.4 ± 5.2	62.9 ± 3.1	66.4 ± 3.4	65.0 ± 3.5	65.0 ± 3.5	64.3 ± 3.2	61.4 ± 3.6
Group B	60.7 ± 6.2	60.7 ± 6.8	60.7 ± 6.2	60.7 ± 6.2	60.7 ± 6.2	65.0 ± 6.1	67.9 ± 5.7

Data are presented as mean ± S.E.

## Data Availability

The data presented in this study are available on request from the corresponding author. The data are not publicly available due to privacy and ethical reasons.
